# Early Care and Education Workers’ Experience and Stress during the COVID-19 Pandemic

**DOI:** 10.3390/ijerph19052670

**Published:** 2022-02-25

**Authors:** Emilee L. Quinn, Bert Stover, Jennifer J. Otten, Noah Seixas

**Affiliations:** 1 Department of Health Systems and Population Health, University of Washington, Seattle, WA 98195, USA; equinn1@uw.edu (E.L.Q.); bstover@uw.edu (B.S.); 2 Department of Environmental and Occupational Health Sciences, University of Washington, Seattle, WA 98195, USA; jotten@uw.edu; 3 Nutritional Sciences Program, University of Washington, Seattle, WA 98195, USA

**Keywords:** COVID-19, early care and education, occupational health, stress, workplace, public health

## Abstract

Early care and education (ECE) workers experience many job-related stressors. During the COVID-19 pandemic, ECE programs either closed or remained open while workers faced additional demands. We deployed a survey of the center-based ECE workforce in Washington State (United States) one year into the COVID-19 pandemic to assess impacts and workers’ perceived stress levels. We describe the prevalence of reported impacts, including workplace closures; job changes; COVID-19 transmission; risk factors for severe COVID-19; the use of social distancing practices; satisfaction with workplace responses; perceptions of worker roles, respect, and influence; and food and financial insecurity. Themes from open-ended responses illustrate how workers’ jobs changed and the stressors that workers experienced as a result. Fifty-seven percent of ECE workers reported moderate or high levels of stress. In a regression model assessing unique contributions to stress, work changes that negatively impacted home life contributed most to stress. Feeling respected for one’s work and feeling positive about one’s role as an “essential worker” contributed to lower levels of stress. Experiencing financial insecurity, caring for school-aged children or children of multiple ages, being younger, and being born in the United States also contributed to higher stress. Findings can inform policies designed to support the workforce.

## 1. Introduction

Early care and education (ECE) workers comprise a critical workforce that was especially vulnerable during the novel coronavirus pandemic. The sector plays an important role in the economy and in the development of young children [1,2]. Despite the vital role of ECE workers, the predominantly female workforce earns some of the lowest wages of all job sectors, receives minimal benefits, experiences high rates of turnover, and is more likely than the general population to rely on safety net programs such as Medicaid [3,4,5,6]. The ECE workforce also faces relatively high exposure to infectious disease and high rates of poor physical and mental health [4,7,8,9,10].

In 2020, the coronavirus disease (COVID-19) pandemic brought these dynamics to the national forefront [11]. Many ECE programs were forced to close due to the combined effects of decreased demand for care, mandated business closures, and preventative public health regulations that led to reduced revenue and increased costs [12,13]. Other programs remained open and grappled with how to serve children and families with considerable uncertainty and more demands related to meeting the needs of the children in their care [13]. Large numbers of ECE workers were laid off across the country; national data showed that the size of the workforce decreased by 35% between February and April 2020, and remained 16% lower a full year later [14].

Like other low-wage workers, such as those in food retail and processing, transportation, and other service sectors, ECE workers were considered “essential” throughout the pandemic. Essential workers, on the whole, experienced high rates of economic vulnerability, faced greater exposure to COVID-19, and reflected systemic racial inequities, given that many earned low wages and were people of color [15,16,17]. Research on experiences of essential workers, including assessments of perceived stress by employment type and characteristics, and the role of stress in mediating other poor outcomes, continues to emerge [18,19,20].

A growing body of research demonstrates that, even in typical times, many ECE workers experience a wide array of job-related stressors related to high job demands, limited job control, and minimal job resources [21]. ECE workers report that work-related time pressures, interpersonal dynamics, demands associated with meeting children’s needs, non-teaching tasks, high rates of turnover among their peers, and perceived imbalances between effort and compensation are among the factors that are most influential in terms of their job satisfaction and general wellbeing [22,23]. Factors including age, teaching competence and efficacy, and a chaotic work environment have all been specifically associated with workers’ stress [24]. Many of these stressors, and their accumulation, are associated with increased rates of burnout and turnover, as well as poor mental and physical health [8,24,25,26,27]. Higher stress experienced by workers is also associated with negative outcomes in teacher-child interactions [28,29,30].

Although many of the challenges faced by ECE businesses during the pandemic have been documented, the vast array of potential impacts of the pandemic on ECE workers have been less studied. A small body of early research from the United States reported changes relating to work schedules and responsibilities (e.g., more cleaning and virtual instruction), increased concerns about health and safety, poorer mental health outcomes, and decreased commitment to the ECE profession [13]. Most of these reports did not include the perspectives of workers who had left their jobs during the pandemic, however, and they reflected a range of ECE settings, samples, and methodologies [13]. More research on the breadth and impact of pandemic-related stressors for ECE workers is needed to inform efforts to strengthen the ECE system generally and in preparation for future disruptions.

In March 2020 in Washington State (United States), a statewide stay-at-home order mandated the closure of many businesses, but classified ECE programs as exempt essential businesses that could remain open [31]. Still, the capacity of open childcare programs in the state decreased by nearly half during the following month, before slowly increasing throughout the remainder of the year [32]. Guidance pertaining to ECE health and safety protocols, including social distancing, the use of personal protective equipment, and child-to-teacher ratios, was issued at multiple levels of government and changed frequently throughout the pandemic [33,34]. In this study we aimed to (1) describe the employment, financial, and health impacts experienced by the ECE workforce in Washington State during the coronavirus pandemic, and, (2) explore the relationship between these multifactorial impacts and ECE workers’ stress levels.

## 2. Materials and Methods

We deployed a statewide survey of Washington State’s center-based ECE workforce at a single time point (February to March 2021), approximately one year into the COVID-19 pandemic. The survey was originally designed to assess current working conditions and health among the workforce, but was amended during the initial stages of the pandemic to further explore how work and health was affected by changes in conditions during the pandemic. The survey relied on self-reporting to collect information about individual workers’ personal demographic characteristics; characteristics of their ECE employment; employment impacts related to the coronavirus pandemic; and work-related health exposures, health behaviors, and outcomes, including stress. The survey asked about current conditions and experiences, as well as those experienced over the prior year.

The research team secured contact information with some descriptive data (e.g., position, race) from the state’s ECE workforce registry for all individuals registered as working in a licensed ECE center with children under the age of six years as of January 2021 [35]. Providers registered as working with family childcare homes or school-aged programs were excluded because of differences in job characteristics. The study team emailed a survey invitation to individuals for whom contact information was provided that included a weblink to the survey and all information required for informed consent. Participants could request a hard copy of the survey as an alternative to the online survey and the survey was available in English and Spanish. The study team sent out a maximum of three survey reminders. The survey instrument included questions from validated and reliable tools [21,36,37,38], as well as those drafted specifically for the study with input from ECE stakeholder agencies from Washington State. It took an estimated 30 to 40 min for workers to complete the survey. Survey participants could enter a raffle for a chance to win one of 122 gift cards ranging in value from USD 20 to USD 500.

Before beginning the survey, participants responded to screening questions to confirm they had worked in an ECE center at some point during the prior year with children under the age of six years. Questions within the survey addressed the demographic and health characteristics of ECE workers and their households; characteristics of respondents’ current or most recent ECE work; and pandemic-related impacts experienced over the prior year. Demographic and health characteristics included gender, age, race, ethnicity, country of birth, education level, household income, and various risk factors for severe COVID-19 [39]. Work characteristics included years of ECE experience and the following in relation to the worker’s current or most recent ECE job: their position, the age of the children they cared for, their hourly wage and annual income, hours worked per week, and employer-provided benefits. Pandemic-related impacts included employment status at the time of the survey, any employment separations experienced over the prior year, and reasons for those separations; food and financial security; and changes in health behaviors (e.g., physical activity, alcohol, and food consumption). Respondents who reported that they remained in the same job throughout the year also reported on changes in the nature of their work (e.g., title change, change in pay or the number of children with which they worked), COVID-19 transmission in the workplace, perceptions of the workplace pandemic response, and perceptions of their role as an “essential worker”.

Respondents’ levels of perceived stress, as measured by the ten-item Perceived Stress Scale (PSS-10), served as the dependent variable. The PSS-10 is widely used for assessing “the degree to which respondents found their lives unpredictable, uncontrollable, and overloading” [40,41] (p. 387). Versions of the Perceived Stress Scale have been used in other studies of both childcare worker wellbeing [8,27,42] and COVID-19 pandemic impacts [43,44]. The use of this tool results in a score ranging from 0 to 40, with “low” stress defined as a score from 0–13, “moderate” ranging from 14–26, and “high” ranging from 27–40. The survey also included one open-ended question about how respondents’ work had changed over the prior year. The Washington State Institutional Review Board approved the study protocol. The research team piloted the survey with five ECE workers and had the survey professionally translated into Spanish before deploying.

Using Stata version 14.0, the team conducted descriptive analyses of the variables, including frequencies and mean values for the full sample and stratified by position type (e.g., teacher, administrator, other) and employment status. Mean values of the PSS-10 were calculated by demographic characteristics, ECE work characteristics, experiences with various pandemic impacts, role perception, and aspects of health status. We conducted bivariate analyses to examine the relationships between perceived stress and these variables using chi-squared, ANOVA, and T-tests to test the statistical significance of these relationships where appropriate.

To assess the factors contributing to perceived stress, we conducted a multiple-step regression process. First, we grouped a priori potential stressors into four sets: basic demographics, ECE employment characteristics, COVID pandemic-related events and conditions, and financial and food security. We developed a base model with demographics first, using a step-wise selection with a *p* value for inclusion of 0.1. We then developed one model each for employment characteristics, pandemic response, and economic security measures, each including the base model variables. Again, we used a step-wise procedure to select from among the variables in each category. Finally, we ran one full model, including all variables from the base model and the significant contributors to the three subsequent models. Each model had a different number of subjects included because of missing data; in particular, only those with current employment in ECE answered questions about pandemic workplace responses.

For qualitative data collected via the open-ended survey question, the team reviewed an initial sample of 20% of the responses and developed a codebook based on emerging themes pertaining to (1) changes in work tasks and responsibilities; (2) changes in other work characteristics; and (3) and expressed concerns, challenges, and stressors. Using the codebook, a team member then reviewed and coded all responses using Dedoose (version 9.0.17). Responses were reviewed by code and respondent position type (teachers, administrator, other) to summarize themes.

## 3. Results

Survey invitations were sent to 28,306 workers. Of these, 2442 workers participated and responded to a sufficient number of questions to be included in the analytic sample. ECE worker respondents were slightly more likely than non-respondents to be in an administrator position (20% compared to 10%) and white (66% compared to 59%).

### 3.1. Personal, Household, Employment, and Health Characteristics of Respondents

#### 3.1.1. Personal and Household Characteristics

Almost all ECE worker respondents were female and slightly more than half were younger than 39 years old (Table 1). Seventy-one percent of those responding to the survey question about race identified as white (88% of whom identified as non-Hispanic white), 18% reported a Hispanic ethnicity, and 20% were born outside the United States. Slightly less than half had a Bachelor’s-level education or higher and about half reported an annual household income less than USD 40,000.

#### 3.1.2. Health Characteristics

Twelve percent of respondents reported fair or poor overall health, but more than 70% reported at least one risk factor for severe COVID-19 identified by the US Centers for Disease Control and Prevention [39] (Table 1). Of the eight risk factors assessed, overweight or obesity were most prevalent, followed by asthma and hypertension. More than half of respondents (57%) reported moderate or high stress.

#### 3.1.3. ECE Employment Characteristics

Slightly more than half of respondents reported fewer than 10 years of ECE experience. In their current or most recent ECE job, two-thirds of respondents worked in a teaching position (i.e., Lead, Assistant Teacher, or Aide), one-quarter reported an administrative position (i.e., Director, Assistant Director, or Program Coordinator), and the remaining reported their position as “other” (e.g., cook, owner, substitute teacher, other programmatic role). (Table 1). A plurality of respondents worked with preschool-aged children (30 months to five years old), followed by multiple age groups, toddlers between 12 and 29 months old, and infants younger than one year old. Five percent of respondents worked with children older than five years. Respondents generally worked at least 35 h per week at their ECE job and most earned USD 30,000 or less per year at this job. Among those who reported an hourly wage, 12% reported earning USD 13.50 or less per hour (the state’s minimum wage in 2020); most reported earning between USD 13.50 and USD 18.00 per hour. Most respondents also reported that their ECE employer offered paid vacation and paid sick leave, but fewer than half received health insurance through their ECE employer. Administrators reported more ECE experience, more working hours per week, greater access to paid leave, and annual incomes that were, on average, 50% higher than teachers.

### 3.2. Employment, Financial, and Health-Related Pandemic Impacts

#### 3.2.1. Changes in Employment and Work

Two-thirds of respondents reported that their ECE workplace closed at some point during the pandemic, but only 5% said it had not yet reopened at the time of the survey (Table 2). Thirty percent of respondents reported permanently or temporarily separating from their pre-pandemic ECE employer (e.g., they quit, were laid off or furloughed). Of those, more than three-quarters said the reason related to the pandemic, including the center closing (*n* = 273) or cutting their hours or position (*n* = 249), followed by not feeling comfortable working (*n* = 134), having to care for a family member (*n* = 66), or becoming too sick to work (*n* = 25). About half of respondents who experienced a separation eventually returned to the same or a different ECE job, and administrators were more likely than those in other positions to do so.

Teachers were more likely than administrators or other staff to experience employment separations during the pandemic (see Supplemental Table S1). Those who experienced employment separations were generally younger, reported lower hourly and annual pay rates, had fewer years of ECE experience, were less likely to have an employer that offered paid vacation or sick leave, were less likely to have employer-provided health insurance, and had lower annual household incomes.

A substantial majority of respondents (85%) who remained employed at the same center throughout the pandemic reported at least one change to their job during this period (Table 2). Administrators were more likely than others to report an increase in work hours, whereas teachers were more likely to report a decrease.

#### 3.2.2. COVID-19 Transmission and Pandemic Response in the Workplace

About 16% of respondents reported having tested positive for coronavirus or experiencing COVID-19 symptoms without being tested, whereas 72% reported a positive case within their workplace community (staff, children, or parents of children) (Table 2). Forty percent of respondents reported high concern about contracting COVID-19 at work.

Nearly 60% of respondents reported sometimes or often working with more than 10 children at a time despite the recommendation to limit groups to 10 or fewer as a preventative measure [45]. Forty percent of respondents reported not always working with the same group of children, another recommended best practice [34]; this was slightly less prevalent for teachers than administrators and those in “other” positions.

Between 14% and 24% of respondents reported negative sentiments about aspects of their workplace’s response to the pandemic (see Table 2). Administrators were less likely to express negative sentiments than teachers and other staff. Slightly more than one-quarter of respondents reported that their family life had been negatively impacted by changes in work relating to the pandemic.

#### 3.2.3. Perception of Role

A large majority of respondents (86%) reported they hoped to be working in the ECE field one year into the future (Table 2). When asked to select one of five possible statements that best expressed how they felt about their role as an essential worker, 62% selected a statement expressing pride or gratitude (“I am proud to be able to continue working under these conditions” or “I am just grateful to still be working/earning a paycheck”). The remainder of respondents selected a statement conveying that they felt conflicted, resigned, or upset (“I understand why we are considered essential, but feel conflicted about working”, “I would rather not be working, but I have to earn a paycheck”, or “I am upset that I have to work under these conditions”).

#### 3.2.4. Financial Security Impacts

Sixty percent of respondents reported that they found paying for basics such as food, housing, medical care, and heating at least somewhat hard to do and a comparable percentage reported that this had become more difficult during the pandemic. (Table 2) A third of respondents reported that their household experienced low or very low food security in the prior year, meaning that they were not able to afford the food they needed. Nearly 60% of respondents reported use of at least one safety net program, such as Medicaid, a nutrition assistance or charitable food program, or unemployment insurance. Teachers were more likely than administrators to report these financial insecurity impacts, and workers who reported a temporary employment separation or unemployment at the time of the survey were more likely to report these impacts than those who stayed at the same job throughout the pandemic (see Supplemental Table S1).

#### 3.2.5. Health Behavior Impacts

When asked whether various health behaviors had changed during the pandemic, most respondents reported no change in their alcohol consumption, but slightly more than half reported a decrease in physical activity and 40% reported that their diet had worsened.

### 3.3. Perceived Stress

Respondents who were female, younger than 39 years old, non-Hispanic, born in the United States, and with lower household incomes reported higher levels of stress than their counterparts. Stress levels differed by race, with those identifying as Native Hawaiian or Pacific Islander reporting the highest mean stress level and those reporting an “other” race reporting the lowest levels. Levels of perceived stress are reported in Table 3 by some employment and health characteristics, and by pandemic impacts.

Respondents’ stress levels differed significantly based on their experience of all work-related pandemic impacts examined (Table 3). The biggest differences in reported stress levels occurred between those who felt that work changes had negatively impacted their family life and those who did not (mean PSS 18.4, considered moderate stress, compared to 13.2, which is considered low stress) and those who felt they were not respected for their work and those who felt they were (mean PSS 17.0, considered moderate stress, compared to 12.7, considered low stress).

### 3.4. Contributors to Perceived Stress

Table 4 presents the results of regression models, considering factors contributing to respondents’ perceived stress levels. In the first model, considering differences by demographic characteristics, gender, age, and country of birth contributed significantly to the model, indicating higher stress among women, younger providers, and native-born workers. Variables tested, but not contributing to perceived stress included race, ethnicity, and educational level. In Model 2, which included base model demographic variables and employment-related characteristics, only years of ECE experience and the age of children cared for contributed to the model; those with longer tenure had slightly lower reported stress and those caring for school-age children reported higher stress. Job position, hourly pay, hours worked, annual income, paid vacation, sick leave, and employer-provided health insurance did not contribute significantly.

When testing the contributions of pandemic-related impacts among workers who continued to work, those reporting that work changes had negatively impacted their private life and those reporting increased financial insecurity during the pandemic had higher stress levels (Model 3). To a lesser degree, this was also true for those who experienced one or more risk factors for severe COVID-19. Those who felt respected for their work and those with positive feelings about their role as an “essential worker” (pride or gratitude) had lower stress levels than their counterparts. Negative feelings about workplace responses during the pandemic, having tested positive for or experienced COVID-19 illness, having experienced COVID-19 cases at the workplace, and employment status at the time of the survey (e.g., unemployed, employed but experienced an employment separation) did not significantly contribute to perceived stress in the model. Participants who experienced food insecurity in the previous 12 months or current difficulty paying for basic living expenses had higher perceived stress (Model 4).

When all significant contributors to perceived stress from the four models were combined (Model 5), the gender of the ECE worker no longer significantly contributed to perceived stress and, although tenure in childcare remained negative, it was no longer statistically significant. The effects of the age group served were similar, although with contributions to stress for both those serving school-age children and those caring for children across multiple age groups. The impact of work changes on home life and financial insecurity contributed to higher stress levels, whereas feeling respected and positive feelings about a role as an essential worker contributed to lower stress in the full model. Neither having risk factors for severe COVID-19, nor feeling that basic needs had become harder to pay for during the pandemic, nor food insecurity remained significant in the full model.

### 3.5. Open-Ended Descriptions of Work Changes

A total of 1462 respondents offered comments in response to the open-ended questions about ways in which their work and responsibilities had changed over the prior year. Three broad categories of findings emerged from these comments. First, respondents described new tasks and responsibilities related to preventative measures associated with the pandemic, including cleaning and sanitizing; health screenings; social distancing measures; more handwashing and use of personal protective equipment; and developing, researching, and enforcing new policies and practices. Many of these changes were described as time-consuming and frustrating since they added to, rather than replaced, other responsibilities (see Table 5 for illustrative quotes).

Second, workers described changes to their jobs that resulted from the dramatic reduction in ECE staffing and changes to student enrollment (Table 5). These included taking on different roles or responsibilities with or without a change in title or compensation. Notably, an estimated 10% of all respondents described changes associated with becoming responsible for supporting virtual learning with young children staying home from ECE temporarily or with school-age students being cared for at ECE facilities while their schools were closed. Respondents also described changes to hours or schedules, working with fewer children, and experiencing center closures, quitting, or being laid off or furloughed.

Finally, respondents described an array of challenges and stressors related to these job changes (Table 5). The most commonly described challenges and stressors included feeling overworked and not having access to adequate breaks, leave, and planning time. Workers also described more time-intensive and difficult interactions with families, concerns about contracting COVID-19 in the workplace, and feeling insufficiently supported or undervalued by management, parents, and society more broadly. Some workers highlighted demands associated with providing emotional support for children, and their families—which they described as particularly difficult when they felt unsupported themselves—as well as concerns about the impact of the pandemic on children in their care and how these impacts were manifested in more challenging behaviors. Relatedly, workers described feeling frustrated that they could not provide developmentally appropriate care. Many noted that the work environment felt unpredictable due to the constant change of protocols, schedules, and assignments. A smaller number of workers reported that they felt isolated and disconnected from the center community when working from home and practicing social distancing. Finally, some respondents explicitly reported that work-related changes impacted their home and personal life. In addition to these challenges reported by the sample broadly, administrators discussed demands related to staff and enrollment turn-over and hiring, the need to provide more support to their staff, a sense of burden for keeping everyone safe, and stresses associated with trying to ensure the financial solvency of their center. Many workers reported poignantly about the cumulative effects of all these factors resulting in overwhelm and burnout.

## 4. Discussion

The first aim of this paper was to describe the types and prevalence of impacts experienced by a large sample of ECE workers in Washington State during the coronavirus pandemic between 2020 and 2021. Two-thirds of the workers in our sample experienced temporary or permanent closures of their workplace and 85% reported one or more job alteration to adjust to the pandemic; in particular, those related to the number and age of children they cared for, hours worked, and pay. The percentage of respondents who tested positive for COVID-19 (7%) was slightly higher than the estimated statewide incidence of COVID-19 as of 31 March 2021 as a percentage of the state population (4.7%) [46,47], and many reported high levels of concern about contracting COVID-19 at work. Forty to sixty percent of respondents reported child-to-adult ratios and cohorting practices that conflicted with the recommended best practices at the time and more than 40% reported one or more concerns or disagreements with the extent to which their workplace was instituting or adhering to preventative practices. Moreover, open-ended survey responses illustrated the breadth of ways in which workers’ jobs and responsibilities had changed over the course of the year. Many workers felt they had essentially taken on entirely new jobs or were doing multiple jobs at once without a corresponding change in pay, preparation, or respect, and with higher exposure to risk. These findings align with and add to a review of other ECE workforce studies and reports from the United States conducted as of early 2021 and several studies published since then [13,48,49,50,51,52].

The second aim of the paper was to assess workers’ stress levels during the pandemic and explore which of the multifactorial potential impacts were most strongly associated with stress. Our findings indicate that the pandemic has exacerbated the stress experienced by ECE workers—particularly those who are younger and least financially secure. Reported stress levels among ECE workers in our sample were higher than those reported in at least one statewide study of ECE workers prior to the pandemic, but lower than one other with a small sample using the same measure [24,42]. Younger workers in our sample reported slightly higher stress levels than older workers and workers born in the United States reported higher stress relative to their foreign-born peers. Furthermore, workers who cared for school-aged children and children of multiple ages reported higher stress levels than those who cared for ages five and younger. This latter finding corroborates our qualitative data and the widespread reporting of ECE workers being asked to care for older children to fill the gap left by K–12 school closures [13]; many workers in this new role felt as if they were doing work they were ill-prepared to do and, in some cases, described resentment because they felt they were doing the work of K–12 teachers who typically get paid more.

Of all the pandemic impacts examined quantitatively, changes in work that negatively impacted workers’ personal lives contributed most to higher stress levels in our model. The link between stress and work-life balance has been reported on previously [53], but the open-ended responses illustrate the multiple ways in which ECE workers experienced this impact during the pandemic, including concerns about being exposed to the novel coronavirus at work and infecting family members, longer work hours and taking work home, working from home, and general exhaustion. Our results also demonstrate that feeling respected and proud or grateful about one’s role as an essential worker is associated with lower levels of stress. The importance of feeling respected for one’s work has also been highlighted in the pre-pandemic ECE workforce literature [54], and pride in or gratitude for one’s role as an essential worker reflects characteristics of intrinsic and external motivating factors that have been shown to be important for job satisfaction in the field [55]. Open-ended responses illustrate the importance of these factors; many workers conveyed that their concerns and value as workers were disregarded by superiors, enrolled family members, and society more broadly—and often noted that although they were classified as “essential”, they did not receive any recognition, support, or hazard pay for this role.

Even accounting for all other contributing factors, workers reporting difficulty paying for basics such as housing, food, and healthcare reported higher levels of stress than those who did not. This is notable, since 60% of the workforce sample reported financial insecurity based on this measure. The impacts of financial insecurity were experienced differently among the ECE workforce, with workers who experienced employment separations during the pandemic or unemployment reporting higher rates of financial hardship, and younger, less experienced workers being most likely to experience employment separations and loss of employment. It is somewhat surprising that, despite findings pertaining to economic insecurity, lower wages and incomes were not associated with higher stress levels; however, other ECE workforce studies have similarly found wages not to be strongly associated with job satisfaction or other wellbeing outcomes, perhaps because other factors confound or mediate the relationship [23,42]. Unfortunately, food and economic insecurity impacts are likely to persist for this workforce unless systemic changes are made; other studies have documented rates of food and financial security at least as high as those identified in this study during non-pandemic times [56,57].

It is notable that some other factors, such as workers’ positions, experience in the field, concerns with workplace responses, and job changes did not contribute to workers’ self-reported stress levels in the full regression model, though years of experience did so in earlier stage of the analysis. Although it seems plausible that experienced workers might have more robust coping mechanisms for multi-tasking or dealing with challenging child behaviors that would reduce stress, other studies have similarly shown these characteristics not to be associated with stress when controlling for other factors [24]. Many respondents reported that they found workplace responses insufficient or unreasonable and described job changes as difficult, so the fact that these impacts did not emerge as significant in the full model may simply reflect the reality that workers faced many stressors during the pandemic. Open-ended responses also alluded to some stressors not assessed quantitatively in our study, such as difficult interactions with families, challenges associated with caring for children spanning different age groups, worry for children’s wellbeing, and burdens associated with providing emotional support to others [21,22]. In particular, workers described the new and outstanding focus on pandemic-related protocols as draining. This theme aligns with prior research showing that significant sources of stress for ECE workers include menial and non-teaching tasks, time pressures, and having to make compromises in their caregiving philosophies [23]. Finally, respondents described feeling overworked and a sense of instability due to significant staff turnover during the pandemic, which aligns with findings from prior studies documenting negative impacts, including increased stress, on ECE workers remaining in high-turnover environments [22,24,26].

Given these findings, the fact that 86% of respondents reported that they hoped to be working in the ECE field in a year is surprising and hopeful. Considerable focus on the ECE workforce during the pandemic has centered on concerns that high demands, limited support, and low pay may lead ECE workers to leave the field permanently, which would have negative impacts for society as a whole [58]. Indeed, administrators in our study described significant difficulties in recruiting and hiring new staff during the pandemic. Although questions about the strength of the profession going forward extend beyond the scope of our study, our findings indicate that many workers are dedicated to the field despite experiencing stress in multiple ways.

These findings can inform recently proposed policy solutions and approaches aimed at improving the health and wellbeing of the ECE workforce in typical times and during public health emergencies such as the COVID-19 pandemic [4,13,59,60]. Specifically, these findings further the case for improved pay and leave policies that support a livable wage so that ECE workers can pay for their basic needs, remain with ECE employers for longer periods of time, and be appropriately compensated for the demands made of them. This change would also improve parity between ECE and other educational sectors and potentially aid in elevating the status of the field, thus conveying respect for the workforce. Our results also indicate that policies aimed at improving access to health care and health promotion supports are needed. Ensuring that ECE workers have affordable employer-provided health insurance is essential if workers are asked to continue in public-facing roles during a pandemic. Access to employee wellness programs could support workers in managing and preventing chronic health conditions which placed them at higher risk of severe COVID-19 during the pandemic. Mechanisms for the timely and adequate distribution of funding, materials, and comprehensible guidance for ECE programs during pandemics or other emergencies (e.g., aimed at reducing transmission and sustaining the programs) are also needed. Public health guidance should be informed by the perspectives of the workforce to ensure that they are feasible within ECE environments. This would reduce the stress and frustration individual programs and their workers experience in trying to “make do” or research their own solutions. Finally, these findings lend support to growing efforts to develop and test interventions aimed at strengthening social support and the individual coping and emotional regulation skills of ECE workers to support worker wellness and foster higher quality care as a result [61].

This study has several notable strengths. First, it uses mixed methods to explore a wide array of possible pandemic impacts, including impacts as described by workers themselves, and uses an analytic approach to highlight which impacts are most strongly related to stress levels. Second, the study involves a large sample and allowed for the inclusion of workers who were both employed and unemployed one year into the pandemic. An official ECE statewide workforce registry comprised the sampling frame and respondents differed in relatively small ways from the broader workforce population with regard to several key demographic characteristics and the proportion experiencing employment separations during the pandemic. Still, the sample likely differs in some important ways from the ECE workforce in other US states or internationally. For instance, based on a 2012 summary of the ECE workforce in the United States, our sample is slightly more likely to work across multiple age groups, has somewhat higher levels of education but fewer years of experience, and a higher hourly wage [62]. Some of these differences may be explained by the timing of data collection during the pandemic.

This study also has several limitations worth noting. First, the cross-sectional design does not allow for the determination of causal relationships. For example, in some cases, job changes may have contributed to higher stress levels, whereas in others, high stress may have led to job changes, such as a decision to leave a job. All data were based on self-reporting and thus may be subject to social desirability bias, though the survey was designed to be confidential in order to address this concern. Furthermore, it is possible that workers who participated in the survey differed in important ways from workers who did not with regard to their experience during the pandemic. In particular, it is notable that ECE administrators from the workforce registry were slightly more likely to respond to the survey than were teachers, and may have been less impacted by the pandemic in some ways. Additionally, given the fluid nature of the pandemic, the data reported here reflect a particular point in time, rather than workers’ experiences of the pandemic as a whole. For example, vaccines had not yet been made available to ECE workers at the time of the survey and that development may have lessened the stress for some workers who were concerned about infection. Finally, given the comprehensive and lengthy nature of the survey, the levels of missing data were higher than ideal for some questions.

## 5. Conclusions

This study adds to the research on the ECE workforce during the COVID-19 pandemic by describing the many impacts workers experienced, as well as workers’ perceived stress. In particular, it is clear that the vast majority experienced at least one of the many cited job changes and a substantial proportion of workers also experienced separation from work and food and financial insecurity. Many reported concerns about exposure to the novel coronavirus, insufficient workplace responses, and various psychosocial impacts. Of all the impacts examined, economic insecurity, a perceived lack of respect, and dissatisfaction about their role as an essential worker, as well as the negative impact of work on their home life, contributed most to worker stress. These findings can inform policies designed to support and sustain the workforce going forward.

## Figures and Tables

**Table 1:** Personal, household, health, and employment characteristics of Washington State early care and education (ECE) worker respondents, February/March 2021 (*n* = 2442).

				% (*n*) ^a^
**Personal and household demographics**				
	Gender			
		Female		95.3 (2259)
	Age			
		<39 years old		50.2 (1222)
	Ethnicity			
		Hispanic, Latino, or Spanish origin		17.9 (427)
	Race			
		White		71.3 (1725)
		Asian		7.3 (177)
		Black		3.6 (86)
		American Indian or Alaska Native		1.7 (41)
		Native Hawaiian or Pacific Islander		1.0 (24)
		Other		9.0 (217)
		Multiple		6.1 (148)
	Country of birth			
		Born in the US		80.2 (1921)
	Education level			
		<High school, GED or high school graduate		28.7 (676)
		Associates degree		25.7 (605)
		Bachelors degree		33.0 (777)
		Graduate or professional degree		12.5 (295)
	Household income			
		<40,000 USD		48.5 (1061)
**Health**				
	Self-reported health			
		Fair or poor		12.2 (296)
		Good, very good, or excellent		87.8 (2131)
	Perceived stress level ^b^			
		Low		43.3 (1045)
		Moderate		52.2 (1259)
		High		4.5 (108)
	Experiences risk factors for severe COVID-19			
		One or more first factors, including:		72.7 (1775)
			Overweight, pregnant excluded	25.6 (540)
			Obese, pregnant excluded	42.8 (903)
			Hypertension	20.2 (478)
			Heart disease or stroke	1.8 (44)
			Diabetes	6.6 (159)
			Asthma	20.3 (489)
			Smoke ≥ 1 cigarette/day	6.1 (146)
			>65 years old	3.6 (88)
			Pregnant	2.2 (50)
**Current or most recent ECE employment**				
	Position			
		Administrator		24.4 (593)
		Teacher		66.4 (1617)
		Other		9.2 (225)
	Years of ECE experience			
		<10 years		54.6 (1327)
	Age of child taught			
		Infants (0 to 11 months)		6.7 (162)
		Toddlers (12 to 29 months)		16.1 (389)
		Preschoolers (30 months–5 years)		43.1 (1042)
		Kindergarten or older		5.2 (126)
		Multiple age groups		28.8 (697)
	Annual income from ECE employer			
		≤30,000 USD/year		53.3 (1205)
	Hourly pay rate			
		≤13.50 USD/hour		11.7 (197)
		$13.51–$18.00/hour		56.9 (960)
		>18.00 USD/hour		31.4 (529)
	Hours worked per week			
		≥35 h/week		79.8 (1928)
	Paid vacation leave offered by ECE employer			
		No		30.0 (733)
	Paid sick leave offered by ECE employer			
		No		21.3 (520)
	Health insurance coverage			
		Through employer		39.0 (952)
		Through another source		51.0 (1244)
		Not covered		10.0 (243)

^a^ Percentage is based on the number of respondents to the question; the number of respondents varied by question. ^b^ Assessed using the 10-item Perceived Stress Scale (PSS-10). Scores range from 0 to 40, with 0–13 classified as “low” stress, 14 to 26 as “moderate” stress, and 27 to 40 as “high” stress.

**Table 2:** Pandemic-related employment and financial security impacts experienced by Washington State early care and education (ECE) worker respondents, February/March 2021.

				All (*n* = 2442) ^a^	Administrator (*n* = 593) ^a^	Teacher (*n* = 1617) ^a^	Other (*n* = 225) ^a^
**Changes in employment**							
	Employer closures during pandemic, % (*n*)						
		Center closed and stayed closed		5.1 (121)	4.1 (24)	5.0 (78)	8.8 (19)
		Center closed and reopened		61.2 (1450)	58 (337)	61.6 (962)	67.7 (147)
		Center never closed		33.7 (797)	37.9 (220)	33.5 (523)	23.5 (51)
	ECE employment status during pandemic, % (*n*)						
		Did not work at ECE center before pandemic		1.1 (27)	0.8 (5)	1.2 (20)	0.9 (2)
		No separation from employer		69.3 (1688)	83.3 (493)	63.5 (1025)	75.0 (168)
		Permanent or temporary separation from employer		29.6 (722)	15.9 (94)	35.3 (569)	24.1 (54)
	Experienced ≥ 1 job change, ^b,c^ % (*n*)			85.1 (1436)	91.7 (452)	82.2 (842)	83.3 (140)
		Change in number of children cared for		79.0 (1309)	85.9 (420)	76.3 (765)	74.4 (122)
			Increased	7.0 (90)	4.3 (18)	8.6 (66)	4.9 (6)
			Decreased	93.0 (1200)	94.8 (398)	89.5 (685)	93.4 (114)
		Change in hours worked		24.6 (594)	37.6 (184)	35.4 (356)	31.9 (53)
			Increased	11.0 (265)	72.3 (133)	30.1 (107)	47.2 (25)
			Decreased	12.7 (305)	26.6 (49)	64.6 (230)	47.2 (25)
		Change in age of children cared for		21.3 (350)	22.0 (107)	21.7 (216)	16.2 (26)
			Older children	44.8 (150)	55.1 (59)	37.0 (80)	38.5 (10)
			Younger children	16.1 (54)	1.9 (2)	23.2 (50)	7.7 (2)
			Wider range of ages	39.1 (131)	40.2 (43)	34.3 (74)	50.0 (13)
		Change in pay		14.7 (354)	19.8 (97)	22.6 (226)	18.3 (30)
			Increased	10.4 (250)	67.0 (65)	70.8 (160)	80.0 (24)
			Decreased	3.0 (72)	25.8 (25)	18.6 (42)	13.3 (4)
		Change in title/position		12.8 (213)	11.0 (54)	13.8 (138)	12.6 (21)
**COVID-19 transmission in the workplace**							
	Tested positive for COVID-19			7.0 (154)	7.1 (39)	7.3 (105)	4.9 (10)
	Experienced symptoms but not tested			8.5 (187)	7.1 (39)	8.8 (126)	9.9 (20)
	Child or adult at center tested positive, % (*n*)			71.5 (1548)	69.2 (394)	72.4 (1011)	71.8 (140)
	Highly concerned about contracting COVID-19 at work, % (*n*)			40.2 (961)	34.0 (200)	43.0 (677)	37.3 (81)
**COVID-19 response in the workplace**							
	More than 10 children in the classroom,^d^ % (*n*)			59.7 (1162)	61.5 (311)	60.5 (771)	48.5 (78)
	Don’t always work with the same group of children,^e^ % (*n*)			40.0 (769)	60.7 (290)	29.6 (383)	65.3 (94)
	Not confident parents adhere to policies/practices,^f^ % (*n*)			24.0 (566)	15.4 (89)	27.1 (421)	24.7 (54)
	Not confident staff adhere to policies/practices,^f^ % (*n*)			18.7 (443)	10.0 (58)	22.1 (346)	17.1 (38)
	Not confident in center policies/practices,^f^ % (*n*)			18.6 (443)	8.3 (48)	22.9 (361)	14.4 (32)
	Disagree with decision to close/open,^f^ % (*n*)			17.3 (410)	8.8 (50)	20.6 (320)	17.9 (39)
	Lack of timely information to share with families,^f^ % (*n*)			14.3 (332)	9.3 (54)	16.5 (251)	12.1 (26)
	Lack of easy access to cleaning materials,^f^ % (*n*)			13.7 (323)	13.6 (79)	14.4 (222)	19.2 (20)
	Lack of easy access to personal protective equipment,^f^ % (*n*)			13.7 (322)	14.1 (82)	14.0 (216)	10.0 (22)
	Changes in work negatively impact private life,^g^ % (*n*)			26.4 (542)	27.4 (152)	26.2 (341)	24.8 (48)
**Perception of ECE role during COVID-19 pandemic**							
	Hope to be working in ECE a year from now, % (*n*)			86.3 (1781)	92.0 (485)	84.3 (1130)	85.2 (161)
	Do not feel respected for my work,^h^ % (*n*)			29.6 (611)	25.1 (139)	32.4 (425)	24.1 (47)
	Do not have influence concerning my work,^i^ % (*n*)			52.7 (1038)	33.0 (180)	61.2 (757)	53.8 (99)
	How I feel about my role as an “essential worker”, % (*n*)						
		Proud or grateful		62.1 (1274)	67.7 (371)	61.1 (785)	61.0 (116)
		Conflicted, resigned, or upset		37.1 (752)	32.3 (177)	38.9 (500)	39.0 (74)
**Financial security impacts**							
	Paying for the basics is hard,^j^ % (*n*)			59.5 (1374)	45.5 (259)	65.0 (999)	51.4 (110)
	Paying for the basics during the pandemic has become harder,^k^ % (*n*)			62.3 (1427)	51.0 (288)	67.3 (1015)	55.9 (118)
	Household experienced food insecurity in prior year, % (*n*)			33.1 (791)	21.6 (127)	37.7 (593)	30.6 (68)
	Household used ≥ 1 safety net program in prior year, ^l^ % (*n*)			59.2 (1445)	47.6 (282)	63.9 (1034)	55.1 (124)

^a^ Percentage is based on the number of respondents to the question; the number of respondents varied by question. ^b^ Respondents could provide more than one response or may have not responded, so percentage totals may not add up to 100%. ^c^ Only respondents who stayed at the same center without a separation could answer this question (*n* = 1688). ^d^ Refers to reports of “often” or “sometimes”; excludes responses of “never”, which was the recommended best practice. ^e^ Refers to reports of “often” or “sometimes”; excludes responses of “always”, which was the recommended best practice. ^f^ Refers to reports of “somewhat disagree” or “strongly disagree” to statements framed in the positive; excludes responses of “somewhat agree” or “strongly agree” or “neither agree nor disagree.” ^g^ Refers to reports of “to a large extent” and “to a very large extent”; excludes responses of “to a very small extent”, “to a small extent”, and “somewhat.” ^h^ Refers to reports of “rarely or never” and “occasionally” excludes responses of “often”, “usually”, and “ most of the time.” ^i^ Refers to reports of “never or hardly ever”, “seldom”, and “sometimes”; excludes responses of “often” or “always”. ^j^ Refers to reports of “very hard”, “hard”, or “somewhat hard”; excludes responses of “not very hard” and “not hard at all.” ^k^ Refers to reports of “somewhat harder” and “very much harder;” excludes responses of “not harder at all.” ^l^ Safety net programs include: Supplemental Nutrition Assistance Program (SNAP), Special Supplemental Nutrition Program for Women, Infants, and Children (WIC), Medicaid, free or reduced-price school lunch, food pantry, and unemployment insurance.

**Table 3:** Perceived stress level of Washington State early care and education (ECE) worker respondents by select employment and health characteristics, and by experience of pandemic-related impacts, February/March 2021.

			Perceived Stress, ^a^ Mean	*n*	*p*-Value
**Current or most recent ECE employment**					
	Position				
		Administrator	14.3 (6.4)	591	0.035
		Teacher	15.1 (6.8)	1589	
		Other	14.7 (6.9)	225	
	Years of ECE experience				
		<10 years	15.8 (6.9)	1309	0.002
		≥10 years	13.7 (6.3)	1087	
	Age of child taught				
		Infants (0 to 11 months)	14.9 (6.7)	151	0.009
		Toddlers (12 to 29 months)	15.2 (7.1)	381	
		Preschoolers (30 months–5 years)	14.4 (6.7)	1034	
		Kindergarten or older	16.5 (6.1)	126	
		Multiple age groups	15.1 (6.7)	694	
	Paid vacation leave offered by ECE employer				
		Yes	14.7 (6.5)	1689	0.071
		No	15.2 (7.2)	723	
	Paid sick leave offered by ECE employer				
		Yes	14.9 (6.7)	1904	0.482
		No	14.7 (7.0)	508	
	Health insurance coverage				
		Through employer	14.6 (6.4)	943	0.422
		Through another source	15.0 (6.9)	1230	
		Not covered	15.1 (7.3)	236	
**Health**					
	Experience ≥ 1 risk factor for severe COVID-19 ^b^				
		No	14.3 (6.8)	658	0.009
		Yes, including:	15.1 (6.7)	1754	
**Changes in employment during pandemic ^c^**					
	Employment status at time of survey				
		Working at same center as before pandemic	14.4	1667	0.018
		Working, but had experienced a permanent or temporary separation	16.0	550	
		Unemployed	15.3	195	
	Experienced job change(s) during the pandemic ^d,e^				
		One or more changes	15.2 (7.0)	991	0.036
		No changes	14.6 (6.5)	1421	
**COVID-19 transmission and response in the workplace ^c^**					
	Believe you experienced illness due to COVID-19				
		Yes, tested positive	15.2 (6.9)	150	<0.001
		Yes, experienced symptoms but wasn’t tested	16.5 (6.8)	186	
		No	14.4 (6.7)	1829	
	One or more person at center tested positive for COVID-19				
		Yes	15.2 (6.6)	1538	<0.001
		No	14.0 (6.9)	609	
	Concern about contracting COVID-19 at center				
		Highly	16.6 (6.6)	948	<0.001
		Not at all or moderately	13.8 (6.6)	1417	
	Disagreement with COVID-19 response in the workplace				
		2 or more disagreements	17.4 (6.7)	586	<0.001
		1 disagreement	15.9 (6.8)	275	
		No	13.5 (6.3)	1324	
	Changes in work have negatively impacted my family/private life				
		To a large or very large extent	18.4 (6.4)	539	<0.001
		To a very small extent, small extent, or somewhat	13.2 (6.1)	1497	
**Financial and food security**					
	Experienced low or very low food insecurity				
		Yes	13.7 (6.5)	1587	<0.001
		No	17.0 (6.7)	780	
	Experienced difficulty paying for the very basics ^f^				
		Yes	16.2 (6.6)	950	<0.001
		No	13.0 (6.4)	1357	
	Experienced more difficulty paying for the very basics during the pandemic				
		Somewhat or very much harder	15.8 (6.7)	1412	<0.001
		Not harder	13.5 (6.4)	862	
	Household used ≥1 safety net program in the last year ^g^				
		Yes	15.3 (6.8)	1431	<0.001
		No	14.3 (6.7)	981	
**Perception of role ^e^**					
	Feels respected for work				
		Rarely/never, occasionally, often	17.0 (6.3)	608	<0.001
		Usually, most of the time	12.7 (6.2)	1437	
	Has a large degree of influence concerning work				
		Never or hardly ever, seldom, sometimes	15.5 (6.5	1032	<0.001
		Often, always	13.7 (6.5)	930	
	Feelings about role as an “essential worker” during the pandemic				
		Conflicted or upset	16.8 (6.4)	750	<0.001
		Proud or grateful	13.2 (6.3)	1261	

^a^ Assessed using the 10-item Perceived Stress Scale (PSS-10); total scores ranged from 0 (low) to 40 (high). ^b^ Accounts only for risk factors including overweight or obese (pregnant excluded), hypertension, heart disease or stroke, diabetes, asthma, smoking ≥ 1 cigarette per day, age of 65 years old or older, and pregnant. ^c^ Based on current or most recent ECE employment. ^d^ Examined changes included changes in title, age or number of children cared for, pay, and hours. ^e^ Only respondents who reported staying at the same center throughout the pandemic without a separation answered this question. ^f^ Includes those who reported paying for the very basics to be “hard,” “very hard,” or “somewhat hard” as opposed to “not very hard” or “not hard at all. ^g^ Safety net programs include Supplemental Nutrition Assistance Program (SNAP), Special Supplemental Nutrition Program for Women, Infants, and Children (WIC), Medicaid, free or reduced-price school lunch, food pantry, and unemployment insurance.

**Table 4:** Effects of personal characteristics, employment conditions, pandemic impacts, and food and financial security on perceived stress among Washington State early care and education (ECE) worker respondents, February/March 2021.

			Model 1 (Base): Demographic Characteristics ^a^	Model 2: Early Care and Education (ECE) Employment Characteristics ^a^	Model 3: Pandemic-Related Impacts ^a^	Model 4: Food and Financial Security ^a^	Model 5: Combined Final Model ^a^
*n*			2306	2266	1737	2174	1674
**Demographic characteristics ^b^**							
	Gender (reference: female)		1.65 (0.64) ***	1.99 (0.65) ***	0.89 (0.69)	0.92 (0.63)	1.06 (0.70)
	Age (years old, continuous)		−0.14 (0.01) ***	−0.12 (0.01) ***	−0.10 (0.01) ***	−0.12 (0.01) ***	−0.09 (0.01) ***
	Country of birth (reference: US-born)		−1.63 (0.34) ***	−1.79 (0.35) ***	−1.20 (0.37) ***	−1.87 (0.34) ***	−1.16 (0.38) ***
**ECE employment characteristics**							
	Years of ECE experience (continuous)			−0.04 (0.02) **			−0.02 (0.02)
	Age of child taught (reference: Preschoolers, 30 months to 5 years)						
		Infants (0 to 11 months)		0.26 (0.58)			0.06 (0.59)
		Toddlers (12 to 29 months)		0.08 (0.40)			0.03 (0.43)
		Kindergarten or older		1.65 (0.63) ***			1.04 (0.63) *
		Multiple age groups		0.48 (0.33)			0.71 (0.33) **
**Pandemic-related impacts**							
	Changes in work during pandemic negatively impacted family/private life (reference: never/hardly ever, seldom, sometimes)				3.44 (0.33) ***		3.4 (0.34) ***
	Usually or most of the time feels respected for work (reference: rarely/never, occasionally, often)				−3.25 (0.32) ***		−3.03 (0.33) ***
	Often or always has a large degree of influence concerning work (reference: never or hardly ever, seldom, sometimes)				−0.38 (0.28)		−0.42 (0.29)
	Experienced ≥1 risk factor for severe COVID-19 (reference: No)				0.58 (0.32) *		0.45 (0.33)
	Feels proud or grateful about role as an “essential worker” during the pandemic (reference: Conflicted or upset)				−1.43 (0.30) ***		−1.39 (0.31) ***
	Experienced more difficulty paying for the very basics during the pandemic (reference: No)						
		Somewhat more			0.52 (0.30) *		−0.26 (0.36)
		Very much more			1.35 (0.44) ***		0.13 (0.55)
	Experienced one or more job changes in prior year (reference: No changes)				0.37 (0.30)		0.35 (0.31)
**Food and financial security**							
	Experienced difficulty paying for the very basics (reference: No)					1.90 (0.31) ***	1.28 (0.37) ***
	Experienced low or very low food security (reference: No)					1.87 (0.32) ***	0.42 (0.37)

^a^ Statistical significance indicated as: * *p* < 0.1, ** *p* < 0.05, *** *p* < 0.01. ^b^ These three demographic variables were included in all models regardless of *p*-value.

**Table 5:** Themes and illustrative quotes regarding work changes in the pandemic among Washington State early care and education (ECE) worker respondents, February/March 2021.

Theme		Illustrative Quotes
Workers faced many new **pandemic-related responsibilities**, including much more cleaning, sanitizing, and handwashing; health screenings; social distancing and the use of personal protective equipment; and developing, researching, and enforcing new policies and practices.		“The increased cleaning is necessary but very time consuming and I worry about the constant exposure to breathing in bleach daily.” (Administrator) “Having to enforce mask wearing and social distancing with small children is a constant challenge and it is not always successful.” (Teacher) “SO MUCH retrieving of children from the school so parents aren’t traveling the halls and rooms of the school. This is the single-most biggest time-waste and interference to our center’s admin staff. We don’t have money to [hire] extra staff, so we just have to work extra.” (Administrator)
Workers experienced many **other job changes** due to changes in staffing and student enrollment: changes to titles and roles; working from home; transition to virtual learning and supporting school-age children; changes in hours; working with fewer children; center closures; and lay-offs, furloughs, or voluntary separations.		“Working from home since June. Working harder and longer hours to guide the center through COVID” (Administrator) “Adding the tasks involved with the older kids virtual schooling increased my responsibility immensely.” (Teacher) “We are working only 25 h a week and we used to work 40. Financially losing around $1000.00 a month…” (Teacher) “I got laid off due to COVID because their weren’t enough children going to the daycare and my boss couldn’t pay me to work.” (Teacher)
**Stressors and challenges** resulting from changes:		
	Feeling overworked and without access to adequate breaks, leave, and planning time	“I went from working with two other teachers to take care of 24 kids to now having 10 3–5 year old’s by myself every day with no break.” (Teacher) “Also, we have numerous staffing/coverage issues, so many of us have to do planning and other prep. work outside of working hours (unpaid). Breaks are usually condensed into one period during the day, instead of spaced out.” (Teacher)
	More time-intensive and difficult interactions with families due to limited in-person opportunities and the need to enforce policies	“Teachers have to communicate via instant messenger or email. It has made building relationships with parents a little challenging.” (Teacher) “I have to deal with parents who do not want to follow the guidelines on a regular basis and am often yelled at and belittled for trying to keep them, their child/children, other staff, and myself safe and healthy during this crisis.” (Administrator)
	Concern about contracting COVID-19 given the nature of ECE work, personal health circumstances, and insufficient adherence to preventative protocol in some cases	“The stress of children coming in sick has been more so and with one year olds, most symptoms are so common from little colds to teething that it is difficult to tell if a child is a ‘risk’ or not.” (Teacher) “More anxiety about being exposed to illness and how being ill will lessen my hours and pay” (Teacher) “The thought of bringing COVID to your own family.” (Administrator)
	Feeling unsupported based on a lack of needed supplies and guidance, and disrespected and undervalued by management, parents, and/or broader society	“As a director I have to make important decisions with little to no guidance because of the nature of the pandemic” (Administrator) “A lot of the staff feel very unappreciated as the community appreciates medical and grocery workers but we get over looked. We have been opened this entire time but we do not even get healthcare benefits or any hazard pay.” (Administrator)
	The need to provide additional emotional support to children, family, and other ECE workers, as well as concern about the impact of the pandemic on children in their care manifesting as behavioral problems	“I have less emotional resources to cope with stress than before and the children are channeling lots of stress, making behaviors more ramped up. Technically we have less children than before but the emotional needs are far greater in the children who are there.” (Teacher)
	Feeling unable to provide high-quality or developmentally appropriate care	“I went from what felt like education in 2019 to being told in 2020 that my job is to simply ensure the parents are happy and keep paying the center and that the kids are supervised” (Teacher) “We can’t have large group activities, we aren’t supposed to let the kids share toys. It feels development ally inappropriate.” (Teacher)
	Work environment feels unpredictable and uncertain due to staffing shortages, changes to guidance, or reasons that are not communicated	“Constant change of direction, polices, decision, and or without clear communication.” (Other) “A lot of changes and added stress moving children around to different classrooms. No consistent schedules. I don’t know when I start, end or have lunch until the day before and then it will change the day of.” (Teacher)
	Feeling isolated and disconnected from the center community when working from home and practicing social distancing	“I have spent an increasing amount of time alone (everyday all day alone) with students which is much more difficult then with a assistant teacher.” (Teacher) “Separated from other staff/teaching assistants -- feels pretty lonely. Can’t interact with parents anymore due to COVID. Really miss those interactions and chances to touch base.” (Teacher)
	Impacts on workers’ personal life (e.g., schedule changes, overwork, loss of professional boundaries, exhaustion, risk)	“Because my full-time job is so exhausting I have no energy after work and often cry” (Teacher) “Increased responsibility taking me away from my children at home that are in need of behavioral support. (single mom, sole custody)” (Other)
	Administrator-specific concerns and stressors related to staff and enrollment turn-over, supporting staff, and ensuring the solvency of the center	“As an owner, my responsibilities have shifted to getting to find enough staff, trying to keep everyone healthy, and trying to maintain even when all is crashing. We closed one site and lost 40 childcare spots. The budget is a constant panic.” (Administrator) “I have all employees, children’s and families responsible on my shoulder” (Administrator)
	The cumulative effects of these things are very stressful and result in feeling overwhelmed and burnt-out.	“More responsibilities, more work, more stress, no support, same amount of money (not a lot), more worry of personal safety and safety of others. The stress and demand to keep all safe is overwhelming.” (Administrator) “I have to take temps, do the COVID checklist, receive babies from the outside door and still be the teacher/caregiver/nurse/secretary/janitor/customer service representative. I literally cry when I’m driving to work at 5 am. It’s too much for any human being.” (Teacher) “I love my job and what I do. But the amount of extra emotional support to the kids and families as well as the constant fear of contracting COVID and being expected to do more by the public/government while they don’t support us financially takes a toll.” (Teacher)

## Data Availability

The data are not publicly available because such use was not authorized by our data-sharing agreement with WA State DCYF or approved by the reviewing institutional review board.

## Supplementary Materials

The following are available online at https://www.mdpi.com/article/10.3390/ijerph19052670/s1, Table S1: Number and percentage of early care and education (ECE) workforce survey respondents by characteristics and pandemic employment status, Washington State, February/March 2021.
