# Supplementary material for: Early Care and Education Workers’ Experience and Stress during the COVID-19 Pandemic
Source: Int J Environ Res Public Health. 2022 Feb 25;19(5):2670. doi: 10.3390/ijerph19052670 (PMC8910108; doi:10.3390/ijerph19052670)
Supplement: Supplementary file 1 [file ijerph-19-02670-s001.zip › ijerph-1590263-supplementary.pdf]

Table S1. Number and percentage of early care and education (ECE) workforce survey respondents by characteristics and pandemic employment status, Washington State, February/March 2021

	Stayed at same job throughout pandemic [a] (n=1,688)	Working after a separation during the pandemic [a,b] (n=554)	Currently unemployed [a] (n=200)	P-value
**Most recent ECE employment characteristics**				
Position				
Administrator, % (n)	29.2 (493)	14.5 (80)	10.2 (20)	<.001[c]
Teacher, % (n)	60.8 (1025)	78.6 (434)	80.2 (158)	
Other, % (n)	10.0 (168)	6.9 (38)	9.6 (19)	
< 10 years of ECE experience, % (n)	49.3 (827)	67.0 (368)	67.0 (132)	<.001[c]
≤ $30,000/year ECE income, % (n)	46.0 (714)	66.6 (349)	77.2 (142)	<.001[c]
Hourly pay rate, mean (SD)	16.64 (2.2)	16.06 (2.4)	15.67 (2.4)	<.001[d]
ECE employer offers paid vacation leave, % (n)	74.7 (1261)	58.5 (324)	62.0 (124)	<.001[c]
ECE employer offers paid sick leave, % (n)	83.4 (1408)	69.7 (386)	64.0 (128)	<.001[c]
Health insurance coverage, % (n)				
Through employer	47.9 (808)	25.5 (141)	1.5 (3)	<.001[c]
Through another source	43.3 (730)	62.8 (347)	83.5 (167)	
Not covered	8.8 (148)	11.8 (65)	15.0 (30)	
**Personal demographic characteristics**				
Female, % (n)	95.5 (1,572)	95.4 (507)	93.3 (180)	.371[c]
Age in years, mean (SD)	40.9 (12.6)	37.5 (13.8)	39.3 (14.0)	.009[d]
Hispanic, Latino, or Spanish origin % (n)	18.4 (303)	15.3 (83)	21.5 (41)	.107[c]
Race, % (n)				
White	72.3 (1205)	72.9 (400)	61.2 (120)	n/a[e]
Asian	7.2 (121)	5.8 (32)	12.2 (24)	n/a[e]
Black	3.2 (53)	3.6 (20)	6.6 (13)	n/a[e]
American Indian or Alaska Native	2.1 (35)	0.6 (3)	1.5 (3)	n/a[e]
Native Hawaiian or Pacific Islander	1.1 (19)	0.6 (3)	1.0 (2)	n/a[e]
Other	9.1 (152)	8.0 (44)	10.7 (21)	n/a[e]
Multiple	5.3 (88)	8.6 (47)	6.6 (13)	n/a[e]
Born in the US, % (n)	80.4 (1,331)	82.7 (449)	72.3 (141)	.007[c]
< High school, high school, or Associates degree, % (n)	54.4 (880)	53.8 (295)	57 (106)	.935[c]
**Economic and household characteristics**				
Household income < $40,000/year, % (n)	45.1 (683)	56.6 (287)	54.5 (91)	<.001[c]
Number of people in household, mean (SD)	3.2 (1.4)	3.0 (1.3)	3.2 (1.4)	.056[d]
Provide direct care for ≥ 1 person at home, % (n)	49.5 (815)	46.3 (253)	47.4 (90)	.392[c]
**Health-related characteristics**				
Self-reported fair or poor health, % (n)	12.4 (73)	12.0 (193)	13.0 (29)	.913[c]
Experienced ≥ 1 risk factors for severe COVID-19, % (n)	73.3 (1237)	72.7 (403)	67.5 (135)	.222[c]
**Financial and food insecurity**				
Difficulty paying for the very basics in the past year, % (n)	22.9 (372)	34.2 (181)	33.7 (59)	< .001[c]
Low or very low food security in the past year, % (n)	30.5 (507)	39.3 (212)	37.5 (72)	< .001[c]
Harder paying for the very basics since COVID-19 pandemic began, % (n)	57.1 (908)	71.5 (378)	82.0 (141)	< .001[c]
Household used ≥ 1 safety net program in last year, [f] % (n)	53.0 (895)	72.9 (404)	73.0 (146)	< .001[c]

[a] Percent is based on number of respondents to the question; number of respondents varied by question.

[b] Includes people that returned to the same job and those that returned to a different ECE job.

[c] Based on a chi-square test

[d] Based on an ANOVA test

[e] Cell counts are too small to compute statistic reliably

[f] Safety net programs include Supplemental Nutrition Assistance Program (SNAP), Special Supplemental Nutrition Program for Women, Infants, and Children (WIC), Medicaid, Free or reduced price school lunch, food pantry, and unemployment insurance
